# A novel risk score based on immune-related genes for hepatocellular carcinoma as a reliable prognostic biomarker and correlated with immune infiltration

**DOI:** 10.3389/fimmu.2022.1023349

**Published:** 2022-10-24

**Authors:** Meiying Long, Zihan Zhou, Xueyan Wei, Qiuling Lin, Moqin Qiu, Yunxiang Zhou, Peiqin Chen, Yanji Jiang, Qiuping Wen, Yingchun Liu, Runwei Li, Xianguo Zhou, Hongping Yu

**Affiliations:** ^1^ Department of Experimental Research, Guangxi Medical University Cancer Hospital, Nanning, Guangxi, China; ^2^ Department of Epidemiology and Health Statistics, School of Public Health, Guangxi Medical University, Nanning, Guangxi, China; ^3^ Department of Cancer Prevention and Control, Guangxi Medical University Cancer Hospital, Nanning, Guangxi, China; ^4^ Department of Clinical Research, Guangxi Medical University Cancer Hospital, Nanning, Guangxi, China; ^5^ Department of Respiratory Oncology, Guangxi Medical University Cancer Hospital, Nanning, Guangxi, China; ^6^ Scientific Research Department, Guangxi Medical University Cancer Hospital, Nanning, Guangxi, China; ^7^ Department of Environmental and Occupational Health, School of Public Health, Indiana University, Bloomington, IN, United States; ^8^ Key Laboratory of Early Prevention and Treatment for Regional High-Frequency Tumor (Guangxi Medical University), Ministry of Education, Nanning, Guangxi, China

**Keywords:** hepatocellular carcinoma, immune-related genes, prognosis model, immune infiltration, risk score

## Abstract

**Background:**

Immunological-related genes (IRGs) play a critical role in the immune microenvironment of tumors. Our study aimed to develop an IRG-based survival prediction model for hepatocellular carcinoma (HCC) patients and to investigate the impact of IRGs on the immune microenvironment.

**Methods:**

Differentially expressed IRGs were obtained from The Genomic Data Commons Data Portal (TCGA) and the immunology database and analysis portal (ImmPort). The univariate Cox regression was used to identify the IRGs linked to overall survival (OS), and a Lasso-regularized Cox proportional hazard model was constructed. The International Cancer Genome Consortium (ICGC) database was used to verify the prediction model. ESTIMATE and CIBERSORT were used to estimate immune cell infiltration in the tumor immune microenvironment (TIME). RNA sequencing was performed on HCC tissue specimens to confirm mRNA expression.

**Results:**

A total of 401 differentially expressed IRGs were identified, and 63 IRGs were found related to OS on the 237 up-regulated IRGs by univariate Cox regression analyses. Finally, five IRGs were selected by the LASSO Cox model, including *SPP1*, *BIRC5*, *STC2*, *GLP1R*, and *RAET1E*. This prognostic model demonstrated satisfactory predictive value in the ICGC dataset. The risk score was an independent predictive predictor for OS in HCC patients. Immune-related analysis showed that the immune infiltration level in the high-risk group was higher, suggesting that the 5-IRG signature may play an important role in mediating immune escape and immune resistance in the TIME of HCC. Finally, we confirmed the 5-IRG signature is highly expressed in 65 HCC patients with good predictive power.

**Conclusion:**

We established and verified a new prognosis model for HCC patients based on survival-related IRGs, and the signature could provide new insights into the prognosis of HCC.

## Introduction

Primary liver cancer is the third most common cause of cancer mortality worldwide (1). Hepatocellular carcinoma (HCC) is the most common type, accounting for approximately 75% of liver cancers (2). Despite significant advancements in HCC diagnosis and treatment, the 5-year survival rate remains poor (3). The most frequent scoring methods used to predict the prognosis of HCC patients and aid in the selection of treatment strategies include TNM staging, liver function prediction, and other scoring systems (4, 5). However, these conventional predictors are non-specific (6, 7) with unstable predictive power. Furthermore, the severity of the disease cannot be determined accurately since clinical indicators are hard to detect in most HCC patients in the early stages (8). Therefore, establishing reliable molecular biomarkers is critical for predicting HCC prognosis and treatment.

Immune-related genes (IRGs) might be one such essential mechanism in HCC that deserves attention. Immunogenomic classification can distinguish the immune status of HCC patients, which could impact the prognosis of the patients with HCC (9, 10). A prognostic model was conducted by Li R. et al (11), who reported the IRG-based signature that can enhance the prognostic assessment of non-small cell lung cancer. A prognostic signature based on IRGs can also indicate the survival and immunotherapy response of HCC patients (12). *CMTM7* and *ORM2* as IRGs, *CMTM7* acts as a tumor suppressor by inhibiting cell cycle progression in liver cancer (13), and *ORM2* is closely associated with cancer-promoting pathways for liver cancer (14). Previous studies have confirmed that the tumor immune microenvironment (TIME) affects the promotion of immune tolerance and escape through various mechanisms and will affect the efficacy of ICIs (15, 16). Evidence suggests that the molecular mechanism underlying the immunological genomic is critical for the prognosis of HCC patients and their response to therapy (17, 18). IRGs may be biomarkers for predicting the prognosis and treatment response in HCC patients. However, little is known about the role of IRGs in HCC.

Various studies have recently revealed genetic indicators for predicting the prognosis of human tumors. The expression pattern of IRGs has been reported to be linked to the risk of developing HCC in individuals with hepatic cirrhosis (19). In hepatitis B virus (HBV)-related HCC patients, an immune score based on immune cell type shows promise as a possible marker to assess overall survival (OS) (20). Wang WJ et al. (21) developed a prognostic model using survival-related IRG to inform prognosis prediction and immunotherapy for HCC patients. A previous study found that 3 immune-related gene signatures (*LPA, BIRC5 and ROBO1*) could help predict the prognosis of HCC patients (22). Most previous prediction models about IRGs focused on all differentially expressed

genes, not specifically up-regulated genes. However, detecting high-expression markers in real-world clinical testing is easier and more accurate.

The present study aimed to establish a prognostic model by screening survival-related up-regulated IRGs from The Cancer Genome Atlas (TCGA) database and validated the model in the International Cancer Genome Consortium (ICGC) database. Further, we used bioinformatics methods to explore the relationship between the risk score model and immune infiltration. The prognostic model may predict the prognosis as well as provide useful information for selecting more specific immunotherapy in HCC patients.

## Methods

### Data collection

Transcriptome RNA-sequencing data and the clinical follow-up information of HCC patients were downloaded from the Genomic Data Commons Data Portal (TCGA) database and the International Cancer Genome Consortium (ICGC) database. Pretreatment of RNA-sequencing data involved the following: (1) removal of HCC patients without OS; (2) removal of recurring HCC samples; (3) and removal of genes with total counts less than 2 in the RNA-seq analysis. Overall, 371 cases of HCC and 50 nontumor tissues were selected from the TCGA database, while 230 cases of HCC were obtained from the ICGC database for external validation. Next, an immunology database and analysis portal (ImmPort) (https://immport.niaid.nih.gov) was used to identify IRGs.

### Differential gene analysis

Differentially expressed genes between HCC and adjacent non-tumors were identified using the “DEseq2” R package (23) with criteria of (1) false discovery rate (FDR) *p*-value< 0.05 and (2) log_2_ |fold change| > 1.Then, all differentially expressed genes were filtered for IRGs.

### Enrichment analysis and protein-protein interaction networks

The R package “clusterProfiler” was used to conduct a Gene Ontology (GO) and Kyoto Encyclopedia of Genes and Genomes (KEGG) pathway enrichment analysis to investigate the putative biological mechanism of differentially expressed IRGs. In addition, the Protein-Protein Interaction Networks (PPI) was constructed to explore the interactions between IRGs by Cytoscape software. Correlation coefficients > 0.7 and *p*-value of<0.05 was selected as the threshold.

### Construction and validation of the immune‐related signature for HCC

Patients with an OS of less than 30 days were excluded from the prognostic risk model to avoid the effect of irrelevant variables. The training set for the prognostic risk model contained the remaining 343 HCC samples from the TCGA dataset. To screen out the prognosis-related differentially expressed IRGs, univariate Cox regression analysis was used, and IRGs with a *p*-value of 0.05 was chosen for OS prediction. The prognosis-related differentially expressed IRGs were subjected to least absolute shrinkage and selection operator (LASSO) Cox regression analysis using the R “glmnet” package. The risk score model was calculated by weighting the estimated Cox regression coefficients. The prediction model’s risk score was calculated as follows:


$$Risk\;score=\;\sum^{}exprIRG_{i}\;\times \;coef_{i}\;$$

where, *exprIRGi* is the standardized expression value of IRG, and *coefi* is the coefficient of IRG in multivariate Cox regression analysis.

Based on the median threshold for the risk score, the patients were separated into low-risk and high-risk groups. The differences in OS between the two groups were assessed using the Kaplan-Meier technique. The receiver operating characteristic (ROC) curve and the area under the curve (AUC) were used to measure the prediction capacity of the risk model. The external validation was conducted using 230 HCC samples with survival information in the ICGC dataset.

### Clinical value of the prognostic signature

This study explored the clinical value of DEIRGs in the prediction model, the univariate and multivariate cox regression analyses were performed to estimate the independent effect of the risk score on the OS and clinical variables (age, sex, pathological T stage) of HCC patients.

### Immune cell infiltration analysis

The proportions of immune and stromal cells were estimated using the ESTIMATE method (24). The “estimate” and “limma” R packages were used to calculate the immunological and stromal scores for each HCC sample. Variations in the quantity of tumor-infiltrating immune cells were checked with CIBERSORT (25, 26) for 22 categories of immune cells in the low-risk and high-risk groups. The expression of common immune checkpoint molecules was then compared between the low-risk and high-risk groups.

### RNA-sequencing

To validate the expression levels of the model signature genes, we analyzed RNA sequencing data from tumor tissues and paired adjacent normal tissues of 65 HCC patients. Adjacent non-tumor tissues: the area of tissues 1cm~2cm beyond the edge of the lesion site. RNA sequencing was performed by Hepalos Bio. The raw sequencing reads were preprocessed by fastp v0.23.0 (27), and HISAT2 (Hierarchical Indexing for Spliced Alignment of Transcripts) (28) was used to align the transcriptome sequencing Reads to the reference genome, and HTSeq (29) was used for Reads Count calculation. Human samples were obtained from HCC patients in Guangxi Medical University Cancer Hospital after written informed consent was obtained. Patient survival information was obtained through the disease management follow-up system. The study protocol was approved (Approval Number: LW2022118) by the Ethics Committee of the Center of Guangxi Medical University Cancer Hospital.

### Statistical analysis

For differential analysis, the “DEseq2” R package was used. LASSO regression analysis was performed using the “glmnet” R package to decrease OS prediction genes and avoid overfitting. Median survival and survival probability were calculated using the Kaplan-Meier method with the “ survivor” R package.The log-rank test was used to perform the Kaplan-Meier survival analysis. A univariate Cox regression analysis was utilized to identify genes associated with OS, and a multivariate Cox proportional hazard regression analysis was used to generate the prediction model. The prognostic significance of the risk score and other clinical-pathological features were evaluated using univariate and multivariate Cox regression analyses. ANOVA analysis was used to examine expression level in different pathological T stage.The correlation was performed by Pearson correlation analysis. Student t-tests were used to examine differences in the infiltration of immune cells between the two groups. Paired-samples t-test was used to compare the expression levels of the 5-IRGs in HCC tissues and adjacent normal tissues. All statistical tests were considered significant if the *p*-value was less than 0.05. R version 4.0.3 (R Foundation for Statistical Computing, Vienna, Austria) was used to perform all statistical analyses.

## Results

### Screening and identification of IRGs

**Figure 1:**
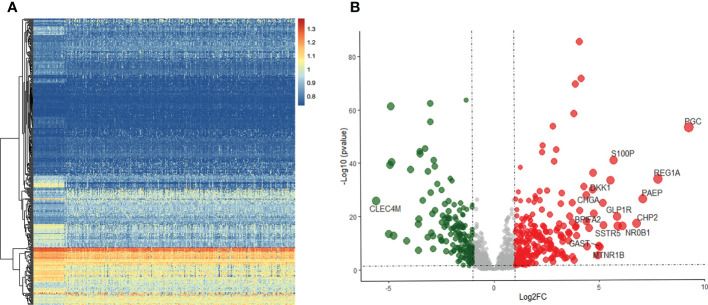
Differentially expressed IRGs in HCC. **(A)** Heatmap of IRGs between HCC and adjacent tissues in TCGA database. **(B)** The volcano plot of differential expression IRGs.

Of the 371 HCC and 50 non-tumor tissue samples studied, 237 genes were up-regulated in tumors, and 164 genes were down-regulated using the specified thresholds of |log FC| > 1 and an FDR p-value< 0.05 (**Figure 1**).

### Function enrichment analysis and PPI

**Figure 2:**
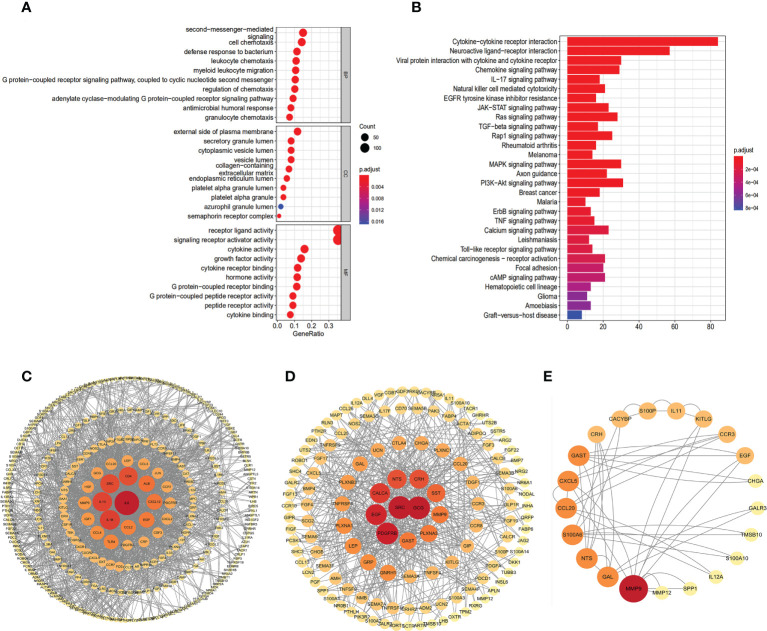
Gene functional enrichment analysis of differentially expressed IRGs. **(A)** The top 30 significant terms of GO function enrichment. BP biological process, CC cellular component, MF molecular function. **(B)** The top 30 significant terms of KEGG analysis.Protein–protein interaction network based on IRGs: all 401 DEIRGs **(C)**, 237 up-regulated IRGs **(D)**, 63 IRGs related to OS **(E)**.

We applied a functional enrichment approach to identify 1,549 GO keywords and 74 significant KEGG pathways to explore the possible practical implications of the 401 differentially expressed IRGs. The dot plot (**Figure 2A**) depicts the top 30 enrichment GO analysis, whereas the barplot (**Figure 2B**) represents the principal 30 enrichment KEGG analysis. “Second-messenger-mediated signaling”, “external side of the plasma membrane,” and “receptor-ligand activity”, were the most abundant GO keywords. KEGG pathway enrichment analyses showed that these genes were associated with signaling pathways relevant to the immune system, including “Cytokine receptor interaction”, “Neuroactive ligand interaction”, and “Viral protein interaction with cytokine and cytokine receptor”. The PPI network clearly illustrates the regulatory relationship between these IRGs (**Figures 2C–E**).

### Establishment of an immune‐related prognostic signature for HCC

**Figure 3:**
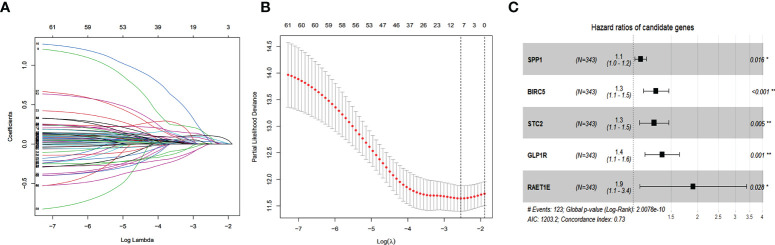
Establishment of Immune-related prognostic signature. **(A)** Screening of optimal parameter (lambda) at which the vertical lines were drawn. **(B)** Lasso coefficient profiles of the seventeen IRGs with non-zero coefficients determined by the optimal lambda. **(C)** The forest plot of multivariate cox analysis to develop a prognostic index based on 5 IRGs.

**Figure 4:**
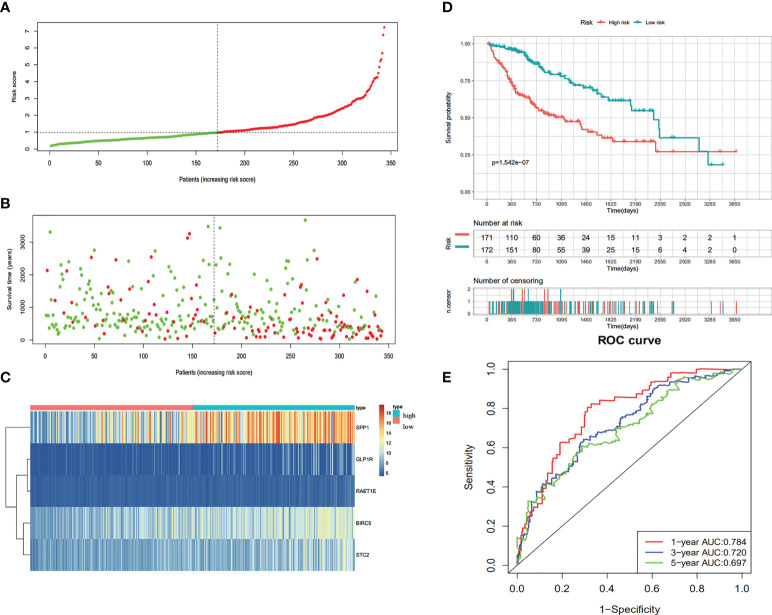
Construction of the immune-based prognostic risk signature in the TCGA cohort. **(A)** The risk score distribution of HCC patients. **(B)** Survival status and duration of patients. **(C)** Heatmap of the expression of the immune-related genes. **(D)** Survival curves for the low risk and high-risk groups. **(E)** Time-independent receiver operating characteristic (ROC) analysis of risk scores for prediction the OS in the TCGA dataset.

According to the univariate Cox regression analysis, 63 of the 237 up-regulated IRGs were considered to have a statistically significant relationship with the OS of HCC patients (**Table S1**). Next, the 63 differentially expressed IRGs were analyzed with LASSO-penalized Cox regression (**Figures 3A, B**). The most significant IRGs were considered risk factors for HCC and used to construct the prediction model (**Table 1**, **Figure 3C**). Based on the median of the risk scores calculated by the prediction model, all patients were divided into high-risk (n = 171) and low-risk (n = 172) groups. The risk score, survival status, and gene expression heatmap are shown in **Figures 4A–C**. According to the Kaplan-Meier analysis, patients in the high-risk group had a far poorer prognosis than those in the low-risk group (**Figure 4D**). The IRGs prognostic signature demonstrated high sensitivity and specificity for predicting the OS with AUC rates of 0.784, 0.720 and 0.697 at 1-year, 3-year and 5-year, respectively (**Figure 4E**).

**Table 1:** Multivariate Cox regression analyses of five IRGs in risk models in HCC.

gene	coef	HR (CI%)	*p*-value
*SPP1*	0.07819	1.0813 (1.0147~1.1524)	0.016
*BIRC5*	0.24337	1.2755 (1.1132~1.4616)	0.000
*STC2*	0.22352	1.2505 (1.0684~1.4635)	0.005
*GLP1R*	0.30847	1.3613 (1.1319~1.6374)	0.001
*RAET1E*	0.64358	1.9033 (1.0705~3.3840)	0.028

### Validation of the immune-related prognostic signature for HCC

**Figure 5:**
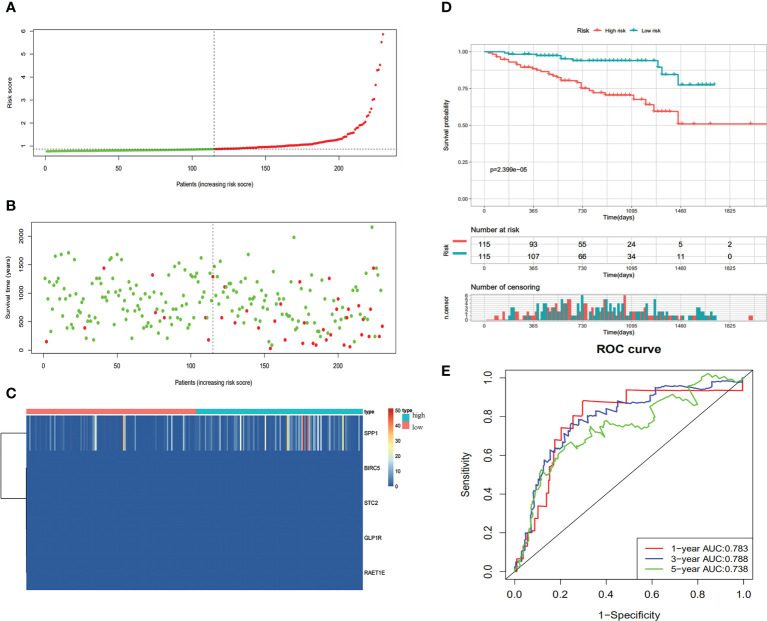
Validation of the immune-based prognostic risk signature in the ICGC cohort. **(A)** The risk score distribution of HCC patients. **(B)** Survival status and duration of patients. **(C)** Heatmap of the expression of the immune-related genes. **(D)** Survival curves for the low risk and high-risk groups. **(E)** Time-independent ROC analysis of risk scores for prediction the overall survival in the ICGC dataset.

We verified the communalism-related signature using an independent verification dataset (ICGC series). According to the median risk score, 230 HCC patients were divided into high-risk (n=115) and low-risk (n=115) groups. Patients in the high-risk group demonstrated significantly poorer OS than those in the low‐risk group (**Figure 5D**). The predictive IRG risk scores, survival status, and gene expression heatmaps are displayed in **Figures 5A–C**. The 1-year, 3-year, and 5-year OS rates had AUCs of 0.783, 0.788, and 0.738, respectively (**Figure 5E**). Therefore, the predictive indicator was found to be trustworthy in the independent verification dataset.

### Clinical value of the prognostic signature

**Figure 6:**
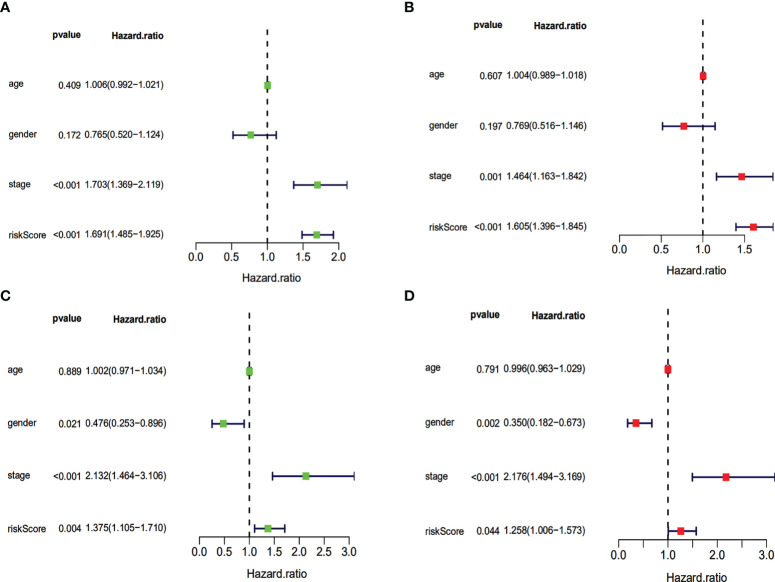
Univariate and multivariate independent prognostic analysis of independent risk factors for OS in patients with HCC. Univariate **(A)** and multivariate **(B)** in the TCGA dataset. Univariate **(C)** and multivariate **(D)** in the ICGC dataset.

**Figure 7:**
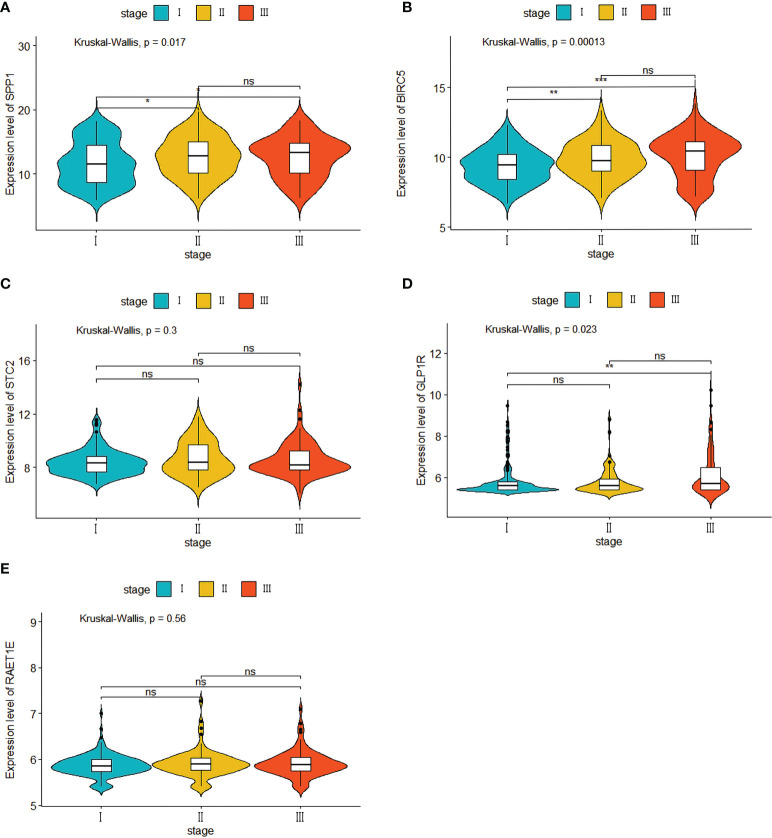
Five IRGs mRNA expression in different pathological T stage in the TCGA cohort. **(A)** the expression of SPP1. **(B)** the expression of BIRC5. **(C)** the expression of STC2. **(D)** the expression of GLP1R. **(E)** the expression of ARET1E. *p<0.05; ** p<0.01; *** p<0.001; ns, not signifcant.

Our evaluation of the independent prediction ability of the 5-IRG risk signature *via* univariate and multivariate Cox regression analyses of signature and other common prognostic factors showed that pathological grade and risk score were associated with OS in the univariate independent prognostic analysis in both datasets (**Figures 6A, B**). Pathological grade and risk score might be independent prognostic factors for survival in HCC patients (**Figures 6C, D**). The expression levels of SPP1, BICR5 and GLP1R differed among the different clinicopathological stages, while STC2 and RAET1E did not(**Figure 7**). Moreover, the overexpression of *SPP1, BIRC5, STC2, GLP1R*, and *RAET1E* was associated with a worse survival rate in HCC patients (**Figure S1**).

### Correlation between IRGs and immune cell infiltration

**Figure 8:**
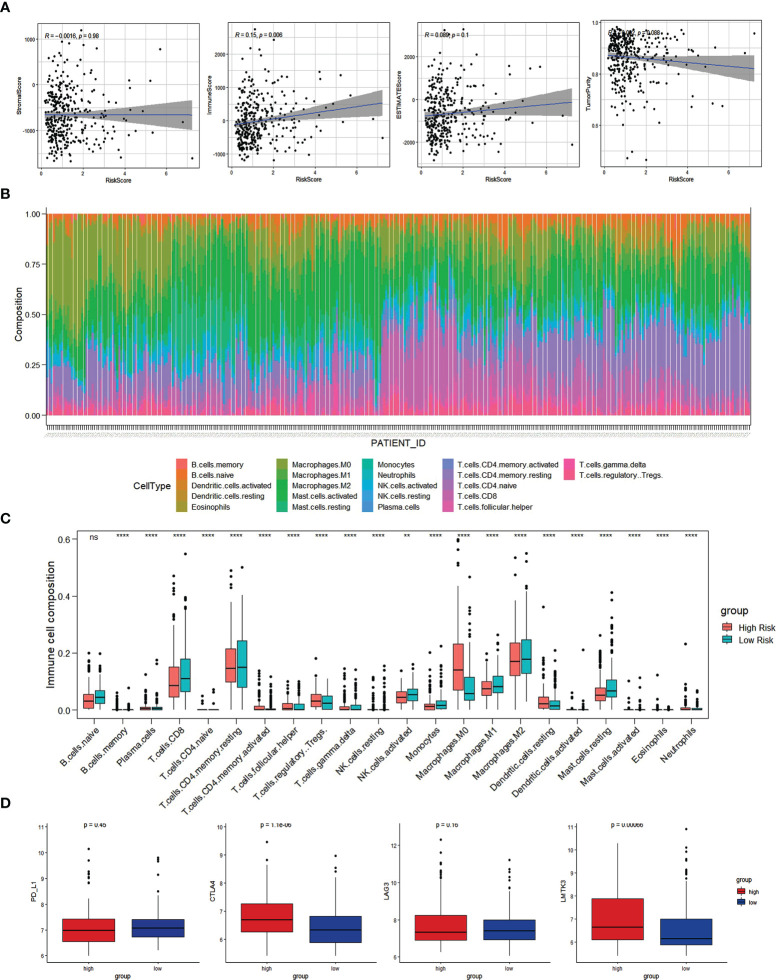
Immune cell infiltration analysis. **(A)** The correlation of Risk score with ESTIMATE analysis in HCC. **(B)** Histogram of the proportion of 22 immune cells in HCC samples. **(C)** The expression of 22 immune cells in low-risk and high-risk groups based on the TCGA dataset. **(D)** Comparisons of immune regulatory molecules in low risk and high-risk groups. *p<0.05; ** p<0.01; *** p<0.001; ns, not signifcant.

Based on the ESTIMATE methodology, we analyzed the capability of the immune-related prognostic signature to predict the TIME by calculating the scores for both immune and stromal cells. According to the results, a higher risk score is associated with a higher immune score (**Figure 8A**). As a follow-up, the CIBERSORT method was used to gather data on the percentage of 22 types of immune cells and then examined the differences between the low-risk and high-risk groups (**Figure 8B**). The infiltration abundance of 22 types of immune cells differed between the risk groups, with M0 macrophages, regulatory T cells, and resting dendritic cells being more abundant in the high-risk group than in the low-risk group, and naive B cells, CD8+ T cells, and activated dendritic cells being less abundant (**Figure 8C**).

### Assessment of the immune checkpoint response in the risk subtypes of HCC patients

HCC samples from low-risk and high-risk groups were compared in terms of expression of the immune checkpoint molecules (*PD-L1, CTLA-4, LAG3, LMTK3*). Compared to low-risk HCC patients, high-risk HCC patients had greater levels of the immune checkpoint molecules, *CTLA-4* and *LMTK3*, indicating that they may be more responsive to therapy using immune checkpoint inhibitors *CTLA-4* and *LMTK3* (**Figure 8D**).

### External sample sequencing validation

**Figure 9:**
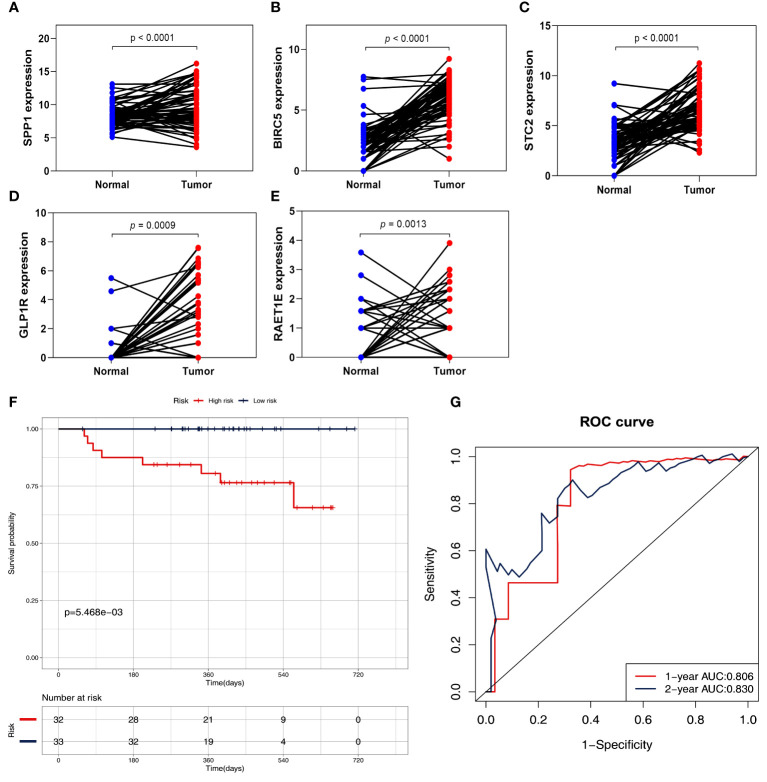
Validation of five IRGs mRNA expression by RNA sequencing in 65 HCC patients. **(A)** the expression of SPP1. **(B)** the expression of BIRC5. **(C)** the expression of STC2. **(D)** the expression of GLP1R. **(E)** the expression of ARET1E. **(F)** Survival curves for the low risk and high-risk groups. **(G)** Time-independent ROC analysis of risk scores for prediction the OS.

To further validate the signature genes, their expression levels were measured using HCC tissues and adjacent normal tissues. The RNA sequencing data based on 65 HCC specimens were validated the given signature set of 5 IRGs (**Figures 9A–E** and **Table S3**). According to the median risk score, the two risk subgroups showed significantly different survival in 65 HCC patients (**Figure 9F** and **Figure S2**), demonstrating the prognostic model’s good discriminatory ability. Meanwhile, the prognostic model presented a good predictive power with AUC rates of 0.806, and 0.830 at 1-year, and 2-year, respectively (**Figure 9G**).

## Discussion

Because the tumor biology of each patient is unique, it is difficult to accurately predict the clinical outcome and immunotherapy response using only a single biomarker. Integrated HCC genomic and transcription data as well as immune response parameters may provide new ideas for the effective prediction of patient prognosis and immune response. Previous research has established that cancer cells regulate the expression pattern of IRGs in healthy cells, thus inhibiting the anti-tumor immune response (30, 31). Tumor cells are exposed to immune cells that regulate IRGs at certain immunological checkpoints. IRGs may serve as new potential biomarkers for HCC prognosis.

In this study, 401 differentially expressed IRGs were screened from the TCGA database, including 237 up-regulated and 164 down-regulated genes. The function enrichment analysis presented the KEGG metabolic pathways as significantly enriched. Considering that the detection of high-expression biomarkers is easier and more accurate in actual clinical testing, we constructed a prediction model based on 237 up-regulated IRGs. Using univariate Cox regression analysis, we identified 63 IRGs that were substantially related to OS. Subsequently, we included the 63 IRGs in the LASSO Cox regression analysis, and the resulting five IRGs were finally included in the prediction model, including *SPP1, BIRC5, STC2, GLP1R*, and *RAET1E*. High expression of the five IRGs in the model resulted in a worse HCC prognosis. HCC patients were classified into two groups (high-risk and low-risk) based on the prediction model’s median risk score.

Dysregulated expression of IRGs may act through metabolic pathways and be involved in hepatocellular carcinoma. A multi-omics study of HCC by Come Hall Z’s team confirmed that specific lipid metabolic pathways are coherently altered when hepatocytes switch to proliferation (32). Aerobic glycolysis acts as a hallmark of hepatocellular carcinoma metabolism and regulates the progression of HCC, such as the PI3K/Akt pathway, AMPK and HIF-1α (33). By modulating epidermal growth factor (*EGFR*) activation, *SPP1* can influence the immune escape and malignant biological activity of tumor cells, and its overexpression enhances HCC development and metastasis (34, 35). *BIRC5*, also known as survivin, is the most effective inhibitor of apoptosis (36), and its high expression in HCC cells promotes proliferation (37). The prognosis of HCC patients with high *STC2* expression is poor, and *STC2* can promote the formation of local blood vessels, tumor proliferation, and metastasis (21, 38). Although the predictive value of *GLP1R* and *RAET1E* in HCC patients has not been reported, they can be used as potential biomarkers. In the verification set, the 5-IRG signature demonstrated strong predictability and repeatability. Our prediction model has a high level of resilience compared to those of other studies (12, 39), and the AUC exhibits excellent discrimination. The model can provide useful prognostic information independently after correcting for other clinical characteristics and might be useful as a potent predicting tool. Consistently, our RNA sequencing analysis revealed that mRNA levels of five IRGs are up-regulated in HCC tissues and that HCC patients with high prognostic features have a poorer OS.

According to the results of clinical trials on ICIs, immune cell infiltration of TIME is a valuable indicator of patient prognosis and the response to immunotherapy (40). ESTIMATE was used to evaluate immune infiltration and found a higher immune infiltration level in the high-risk group, suggesting that the 5-IRG signature may play a key role in mediating the immune escape and immune resistance in the TIME. Among the 22 types of immune cells, Tregs, macrophages M0, dendritic cells, memory CD4 + T cells, and follicular helper cells were more abundant in high-risk patients. It has been reported that tumor-associated neutrophils in HCC can recruit macrophages and Treg cells into the TIME to form an immunosuppressive microenvironment (41–43).In summary, we postulate that the expression pattern of IRGs influences the degree of immune cell infiltration in HCC, hence reducing the antitumor immune response. However, we acknowledge that further experimental verification is required. Immune checkpoints are inhibitory pathways in the immune system, and some immune checkpoint molecules are targets of immunotherapy. Overexpression of the programmed cell death ligand 1 (*PD-L1*) in HCC inhibits the proliferation and activation of T cells, and blocking the *PD-1*/*PD-L1* interaction can enhance immune normalization and the antitumor response (44–47). The anti-*CTLA*-4 monoclonal antibody tremelimumab demonstrates good antitumor activity, both in a single drug and in combination with other drugs (48, 49). Our findings imply that anti-*CTLA*-4 and *LMTK3* antibodies may be an effective treatment for high-risk HCC patients.

This study had some limitations. Firstly, the clinical and pathological information of HCC patients obtained from public databases is limited, which may decrease the predictive power of our model. Secondly, our research was retrospective, so multicenter prospective clinical studies are needed to confirm the model’s predictive potential. The effectiveness of the 5-IRG signature and the mechanism behind the five IRGs remain unclear and require further investigation.

## Conclusion

We established an HCC prognosis prediction model based on five IRGs and verified that the risk score had an excellent predictive performance for the prognosis of HCC patients. The risk score can represent the immune cell infiltration in the TIME, indicating a patient’s immunotherapy response. This prediction model may contribute to more tailored and precise therapy for patients, ultimately resulting in a better patient prognosis.

## Data availability statement

This study obtained mRNA expression profile and clinical data information of HCC from publicly available databases: TCGA LIHC (https://portal.gdc.cancer.gov/) and LIRI-JP of ICGC (https://dcc.icgc.org/). The immune-related genes list was obtained from ImmPort (https://immport.niaid.nih.gov). The RNA sequencing data presented in the study are deposited in the Gene Expression Omnibus (GEO) database, accession number GSE214846.

## Ethics statement

The studies involving human participants were reviewed and approved by Ethics Committee of the Center of Guangxi Medical University Cancer Hospital (Approval Number: LW2022118). The patients/participants provided their written informed consent to participate in this study.

## Author contributions

ML provided the main concepts of this article. ML and ZZ wrote the manuscript text. ML, XW, QL, MQ, and YZ completed the arithmetic analysis. PC and YJ organized the article data results and draw result tables. QW and YL prepared all figures. RL, XZ, and HY reviewed the manuscript. All authors contributed to the article and approved the submitted version.

## Funding

This study was supported by the Key Research and Development Project of Guangxi (Grant Numbers AB18050020 and AA18221001), the Natural Science Foundation of Guangxi Province(Grant Number 2018GXNSFDA050012), the Promoting Project of Basic Capacity for Young and Middle-aged University Teachers in Guangxi (Grant Number 2021KY0099), Shanghai Wu MengChao Medical Science Foundation (Grant Number JJHXM-2019042), the Key Laboratory of Early Prevention and Treatment for Regional High Frequency Tumor(Guangxi Medical University), Ministry of Education (Grant Numbers GKE-KF202007 and GKE-ZZ202118), ʻGuangxi BaGui Scholarsʼ Special Fund.

## Conflict of interest

The authors declare that the research was conducted in the absence of any commercial or financial relationships that could be construed as a potential conflict of interest.

## Publisher’s note

All claims expressed in this article are solely those of the authors and do not necessarily represent those of their affiliated organizations, or those of the publisher, the editors and the reviewers. Any product that may be evaluated in this article, or claim that may be made by its manufacturer, is not guaranteed or endorsed by the publisher.

## Abbreviations

IRGs, immune-related genes; HCC, hepatocellular carcinoma; TCGA, The Cancer Genome Atlas database; OS, overall survival; TME, tumor immune microenvironment; HBV, hepatitis B; ICI, immune checkpoint inhibitors, ROC, receiver operating characteristic curve; AUC, area under the curve; ESTIMATE, Estimation of Stromal and Immune Cells in Malignant Tumor Tissues Using Expression Data.
