# Supplementary figures and images for: A novel risk score based on immune-related genes for hepatocellular carcinoma as a reliable prognostic biomarker and correlated with immune infiltration
Source: Front Immunol. 2022 Oct 24;13:1023349. doi: 10.3389/fimmu.2022.1023349 (PMC9637590; doi:10.3389/fimmu.2022.1023349)

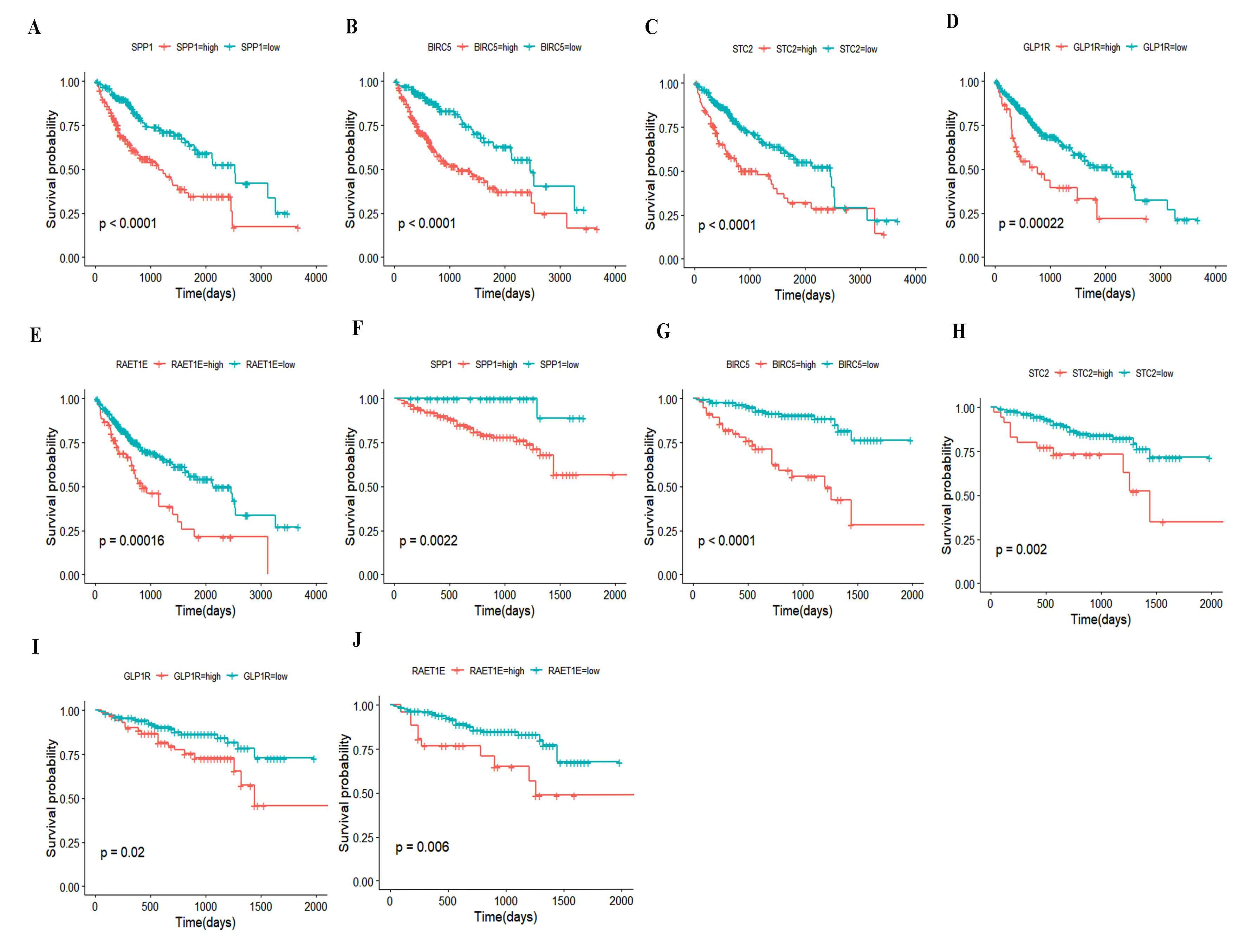

Supplement: Supplementary file 1 [file Image_1.jpeg]

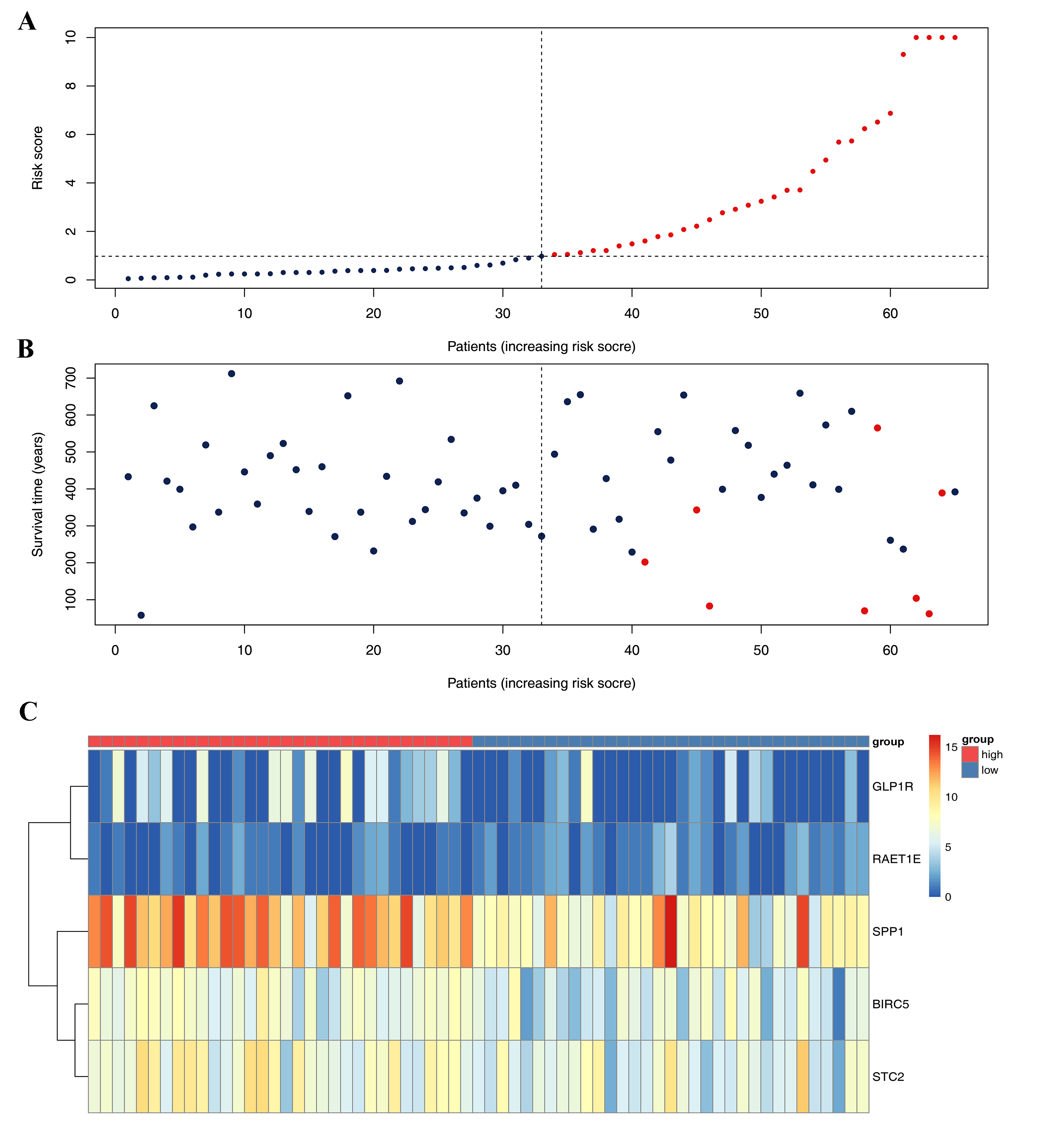

Supplement: Supplementary file 2 [file Image_2.jpeg]
