# Supplementary material for: A novel risk score based on immune-related genes for hepatocellular carcinoma as a reliable prognostic biomarker and correlated with immune infiltration
Source: Front Immunol. 2022 Oct 24;13:1023349. doi: 10.3389/fimmu.2022.1023349 (PMC9637590; doi:10.3389/fimmu.2022.1023349)
Supplement: Supplementary file 3 [file Table_1.docx]

**Table. S1** The top 20 differentially expressed IRGs of Univariate COX regression analysis results

| gene | HR | 95%CI | *p*-value |
| --- | --- | --- | --- |
| *QRFP* | 2.8529 | 1.3270~6.1333 | 0.007 |
| *LCN1* | 2.7378 | 1.0114~7.4111 | 0.047 |
| *RAET1E* | 2.5808 | 1.4031~4.7472 | 0.002 |
| *CRH* | 2.2754 | 1.4582~3.5507 | 0.000 |
| *PGLYRP4* | 2.2103 | 1.4361~3.4017 | 0.000 |
| *LHB* | 2.0374 | 1.2766~3.2516 | 0.003 |
| *PRKCG* | 1.9774 | 1.0985~3.5596 | 0.023 |
| *IL12A* | 1.9292 | 1.2107~3.0743 | 0.006 |
| *CALCR* | 1.7991 | 1.3905~2.3278 | 0.000 |
| *FABP6* | 1.5091 | 1.2576~1.8109 | 0.000 |
| *CACYBP* | 1.4827 | 1.1303~1.9450 | 0.004 |
| *CCR3* | 1.4147 | 1.1599~1.7254 | 0.001 |
| *PLXNA1* | 1.4011 | 1.1634~1.6874 | 0.000 |
| *NR0B1* | 1.3785 | 1.2125~1.5673 | 0.000 |
| *GLP1R* | 1.3769 | 1.1470~1.6527 | 0.001 |
| *ULBP1* | 1.3543 | 1.0026~1.8292 | 0.048 |
| *NR6A1* | 1.3525 | 1.0698~1.7099 | 0.012 |
| *CSPG5* | 1.3505 | 1.0878~1.6766 | 0.006 |
| *STC2* | 1.3409 | 1.1674~1.5403 | 0.000 |
| *GAL* | 1.3323 | 1.0621~1.6713 | 0.013 |
