# Supplementary material for: A novel risk score based on immune-related genes for hepatocellular carcinoma as a reliable prognostic biomarker and correlated with immune infiltration
Source: Front Immunol. 2022 Oct 24;13:1023349. doi: 10.3389/fimmu.2022.1023349 (PMC9637590; doi:10.3389/fimmu.2022.1023349)
Supplement: Supplementary file 4 [file Table_2.docx]

| **KEGG pathway** | **Genes** |
| --- | --- |
| **Cytokine receptor interaction** | CCL23, TNFRSF4, LIFR, IL1RAP, CD4, CXCL12, GHR, CCL25, IL1RL1, IL1RN, IL3RA, IL17D, IL6ST, CCL14, TNFRSF25, CXCL14, TNFSF4, CXCR2, CXCL17, CXCR1, TSLP, ACVR1C, CCR1, TNFRSF18, INHA, CCL3, IL18R1, NGFR, CCL26, IL1B, AMH, AMHR2, BMP10, TNFSF15, IL33, GDF2, BMP7, CSF3, PPBP, CCR3, IL10, BMP4, IL17B, BMP5, TNFRSF19, CXCL2, CCL4, CRLF2, CCL13, INHBC, IL6, CSF1R, CCL2, GDF10, IL2RB, CCR9, BMP8B, TNFSF9, IL5RA, CCR10, NODAL, INHBA, IL12A, TNFRSF9, IL37, BMP8A, GDF5, IL13RA2, IL31RA, CXCL5, CCL20, BMPR1B, TNFSF11, CD70, CCR8, IL11, GDF6, XCR1, IFNL1, PF4, GDF3, LEP, IL31 |
| **Neuroactive ligand interaction** | APLN, PTH1R, VIPR1, GHR, NMB, FPR2, EDNRB, GLP1R, RXFP1, GHRHR, CRHR2, AGTR1, UCN2, SSTR5, FPR1, NTS, PTH2R, UCN, ADM, C5AR1, MC1R, GNRH1, AVPR1A, GLP2R, AVPR2, PTGDR, PTGFR, PTGER2, MTNR1B, GIPR, CALCA, OXTR, GAL, UTS2B, GRP, GALR2, CALCB, ADRB2, MCHR1, ADRB1, GIP, LHB, TACR1, UTS2, GALR3, GCGR, RLN3, SCTR, GCG, SST, OXT, PMCH, CALCR, LEP, EDN3, MLNR |
| **Viral protein interaction with cytokine and cytokine receptor** | CCL23, CXCL12, CCL25, IL6ST, CCL14, CXCL14, CXCR2, CXCR1, CCR1, CCL3, IL18R1, CCL26, PPBP, CCR3, IL10, CXCL2, CCL4, CCL13, IL6, CSF1R, CCL2, IL2RB, CCR9, CCR10, IL37, CXCL5, CCL20, CCR8, XCR1 |

**Table S2** The top3 KEGG pathway gene list
