# Supplementary material for: A novel risk score based on immune-related genes for hepatocellular carcinoma as a reliable prognostic biomarker and correlated with immune infiltration
Source: Front Immunol. 2022 Oct 24;13:1023349. doi: 10.3389/fimmu.2022.1023349 (PMC9637590; doi:10.3389/fimmu.2022.1023349)
Supplement: Supplementary file 5 [file Table_3.docx]

**Table S3** Differential gene analysis of the five IRG in risk models in 65 HCC patients

| gene | log2FC | FDR |
| --- | --- | --- |
| SPP1 | 2.827957017 | 2.17E-16 |
| BIRC5 | 2.846603147 | 2.97E-24 |
| STC2 | 3.398335573 | 1.07E-26 |
| GLP1R | 4.18168477 | 9.98E-07 |
| RAET1E | 0.745088262 | 0.086867296 |
